# Short-term outcomes associated with oral prednisolone versus posterior sub-Tenon triamcinolone acetonide in acute ocular Vogt-Koyanagi-Harada disease

**DOI:** 10.1186/s12886-026-05448-7

**Published:** 2026-10-24

**Authors:** Ehab Mohamed Elsayed Saad, Mohamed I. Abouelela, Mohamed Anany Elsayed

**Affiliations:** Department of Ophthalmology, Faculty of Medicine, Benha University, Benha, Egypt

**Keywords:** Vogt–Koyanagi–Harada disease, Acute uveitis, Oral prednisolone, Posterior sub-Tenon triamcinolone, Optical coherence tomography, Corticosteroid therapy, Recurrence

## Abstract

**Purpose:**

To describe short-term clinical and anatomical outcomes associated with oral prednisolone and posterior sub-Tenon triamcinolone acetonide as initial corticosteroid-based treatment strategies in acute ocular Vogt–Koyanagi–Harada Disease (VKH).

**Methods:**

This retrospective comparative cohort study included 30 treatment-naïve patients (60 eyes) with acute ocular VKH treated at a tertiary referral center between January 2021 and December 2025. Patients were classified using a study-specific two-tier designation (definite or probable VKH) derived from the 2001 Revised Diagnostic Criteria. Treatment assignment (oral prednisolone, *n* = 15, 1.0 mg/kg/day tapered over 3–6 weeks; or PST/STA, *n* = 15) was guided by clinical severity and systemic involvement. The primary outcome was complete clinical and anatomical ocular control at 3 months, defined as absence of clinically active intraocular inflammation, resolution of subretinal fluid on OCT, and stable or improved BCVA. Secondary outcomes included BCVA over 6 months, OCT-based anatomical recovery, recurrence rates, rescue therapy requirement, and adverse events. Resolution of subretinal fluid was assessed qualitatively on OCT as complete disappearance of the subretinal fluid pocket, with central macular thickness (CMT) recorded in parallel as a supportive structural measure. Statistical analyses included the independent and paired t-tests, two-sided Fisher’s exact test, and Kaplan–Meier analysis with the log-rank test.

**Results:**

At 3 months, complete clinical and anatomical ocular control was achieved in 12/15 (80.0%) patients receiving prednisolone and 11/15 (73.3%) receiving PST/STA (*p* = 1.00, Fisher’s exact test). Resolution of subretinal fluid occurred in 86.7% (13/15) and 80.0% (12/15) of patients, respectively (*p* = 1.00, Fisher’s exact test). Mean BCVA improved in both groups over 6 months, with no statistically significant between-group difference. OCT analysis demonstrated no statistically significant between-group differences in the assessed anatomical outcomes. Recurrence occurred in 20.0% of the prednisolone group and 26.7% of the PST/STA group (log-rank *p* = 0.68). Systemic corticosteroid–related metabolic complications were observed only in the prednisolone group, while PST/STA was associated with transient local discomfort and mild intraocular pressure elevation. No serious ocular complications occurred.

**Conclusion:**

In this retrospective, non-randomized cohort, no statistically significant differences were detected between oral prednisolone and PST/STA in the short-term clinical and anatomical outcomes assessed. Because treatment allocation was influenced by disease severity and systemic involvement, the sample size was small, no a priori sample-size calculation was performed, and the systemic corticosteroid regimen was abbreviated, these findings should not be interpreted as evidence of therapeutic equivalence or as establishing PST/STA as an alternative to systemic corticosteroid therapy. Prospective, adequately powered studies with standardized assessment of ocular and systemic disease severity are needed.

**Trial registration:**

ClinicalTrials.gov, NCT07405489. Registered 30 January 2026 (retrospectively registered), https://clinicaltrials.gov/study/NCT07405489.

## Background

Vogt–Koyanagi–Harada (VKH) disease is a rare, multisystem, T cell–mediated autoimmune disorder targeting melanocyte-containing tissues, predominantly affecting the uveal tract, meninges, inner ear, and skin. Acute ocular VKH typically presents as bilateral granulomatous panuveitis, diffuse choroidal inflammation with serous retinal detachments, and optic nerve swelling. Systemic manifestations, including meningismus, tinnitus, and integumentary signs, may develop later in the disease course. If inflammation is untreated or inadequately controlled, VKH may progress to a chronic recurrent phase, leading to fundus depigmentation (“sunset-glow” fundus) and long-term complications such as cataract, glaucoma, and significant visual morbidity [1].

Early and aggressive suppression of ocular inflammation is critical to minimize both short-term tissue damage and long-term sequelae. High-dose systemic corticosteroids, administered orally (e.g., prednisolone 1–1.5 mg/kg/day) or intravenously as pulse therapy, followed by a gradual taper over several months, remain the mainstay of therapy [2]. Prompt initiation and slow tapering, typically over at least six months, reduce recurrence risk and improve visual outcomes. However, systemic corticosteroids carry well-documented adverse effects, including hyperglycemia, hypertension, immunosuppression, cataract, and steroid-induced glaucoma [3].

Local corticosteroid strategies, such as posterior sub-Tenon triamcinolone acetonide (PST/STA) injections, have been explored as primary or adjunctive treatment in acute VKH. Hosoda et al., reported that primary PST/STA treatment in acute VKH achieved complete resolution without recurrence in 21 of 27 eyes (78%), supporting its potential role as a corticosteroid-sparing strategy in selected patients [4]. These periocular injections target ocular inflammation directly, potentially reducing systemic exposure and associated adverse effects. Systemic manifestations in the acute uveitic phase of VKH are variable; integumentary findings may be absent, and neurologic involvement is not universal. Although these findings are encouraging, high-level evidence comparing oral corticosteroids with PST/STA injections in acute VKH is limited. Most prior studies are retrospective, involve small sample sizes, and use heterogeneous treatment protocols [5].

In the present study, patients were classified using a study-specific two-tier designation (definite or probable VKH) derived from the 2001 Revised Diagnostic Criteria, with documentation of acute ocular signs and exclusion of alternative causes. Treatment assignment (oral prednisolone or PST/STA) was guided by clinical severity, ocular and systemic involvement, and physician judgment, following institutional protocols.

Optical coherence tomography (OCT) was used to assess the presence or absence of subretinal fluid qualitatively, with central macular thickness recorded separately as a supportive structural measure. While OCT is not part of standard VKH diagnostic criteria and is not yet validated as a follow-up modality, it provides reproducible visualization of serous retinal detachments and was used to track anatomical resolution [6]. Resolution was defined qualitatively as the complete disappearance of the subretinal fluid pocket on OCT; subretinal fluid height, area, or volume was not quantified. This approach is acknowledged as a study limitation and is discussed accordingly.

Contemporary management of acute initial-onset VKH increasingly emphasizes prompt, aggressive suppression of inflammation and sustained corticosteroid therapy, with early consideration of corticosteroid-sparing immunomodulatory therapy in appropriate patients. This approach aims not only to achieve rapid anatomical recovery but also to prevent persistent subclinical choroidal inflammation and progression toward chronic recurrent disease. Recent literature has highlighted a potential therapeutic window during the initial-onset phase, during which early and sustained treatment may reduce the risk of chronicity and long-term complications.

Nevertheless, systemic corticosteroid therapy may be associated with clinically significant metabolic and other systemic adverse effects. Therefore, the potential role of localized corticosteroid therapy remains clinically relevant in carefully selected patients with predominantly ocular disease and limited systemic involvement. The present study was designed to compare short-term clinical and anatomical ocular outcomes following oral prednisolone versus posterior sub-Tenon triamcinolone acetonide (PST/STA), while recognizing that these treatment strategies do not represent the full spectrum of contemporary VKH management.

This study aimed to describe and compare short-term clinical and anatomical outcomes, recurrence, and observed adverse events associated with these two treatment strategies, using real-world data from a tertiary referral center.

## Patients and methods

This retrospective comparative cohort study described short-term outcomes and adverse events associated with systemic oral prednisolone and PST/STA as initial treatment for acute ocular VKH. The research was performed in the Ophthalmology Department of Benha University Hospital, Egypt, by examining electronic and paper medical records of patients diagnosed from January 2021 to December 2025.

### Study population

Medical records of all patients diagnosed with acute-onset ocular VKH disease during the study period were reviewed. Diagnosis was established according to the 2001 Revised Diagnostic Criteria for VKH disease [7], which define complete, incomplete, and probable VKH based on bilateral ocular findings (granulomatous panuveitis, serous retinal detachment, and choroidal inflammation on fundus examination), the presence or absence of extraocular neurologic, auditory, or integumentary manifestations, and exclusion of alternative causes of bilateral posterior uveitis. For the purposes of this study, patients meeting ocular criteria together with one or more extraocular manifestations (complete or incomplete VKH) were grouped as definite VKH, and patients meeting ocular criteria alone, without extraocular manifestations at presentation or during follow-up, were classified as probable VKH; this two-tier grouping was used because extraocular findings were not graded with sufficient granularity in this retrospective cohort to reliably distinguish complete from incomplete disease on chart review. A formal patient-level breakdown into complete, incomplete, and probable VKH by treatment group could not be reconstructed retrospectively with confidence and is not reported; this is stated as a limitation below. All patients underwent a standard clinical and laboratory work-up at presentation to exclude infectious and other mimickers of bilateral granulomatous panuveitis before a diagnosis of VKH was assigned, consistent with routine uveitis practice at our center. This included syphilis serology (rapid plasma reagin or VDRL with treponemal confirmation), consistent with recommended multimodal-imaging-based exclusion of syphilitic posterior uveitis as a VKH mimicker [8], assessment for tuberculosis exposure, and clinical exclusion of sympathetic ophthalmia (absence of prior ocular surgery or penetrating trauma) and of other causes of bilateral serous retinal detachment. Patients with a positive infectious work-up or an alternative identifiable cause of bilateral posterior uveitis were excluded from the diagnosis of VKH and were not included in this cohort. In addition to diagnostic classification, patients were characterized according to disease stage as acute initial-onset disease or chronic/recurrent disease based on the clinical history and absence or presence of previous VKH episodes. All patients included in the present cohort represented acute initial-onset ocular VKH without a documented previous episode of VKH. Given the early presentation, classification was primarily based on acute ocular features and exclusion criteria. All patients underwent careful systemic evaluation at presentation and during follow-up visits to identify evolving extraocular manifestations, and classification was confirmed accordingly when applicable.

Only treatment-naïve patients who received either systemic oral prednisolone or PST/STA injection as initial therapy were eligible. All patients had bilateral disease, and both eyes of each patient received the same treatment. To avoid inter-eye correlation, the patient was considered the unit of analysis. For continuous ocular variables (best-corrected visual acuity [BCVA], central macular thickness [CMT], and other quantitative OCT parameters), the mean of both eyes was calculated at each visit and used for statistical analysis. For binary anatomical and clinical outcomes (complete clinical and anatomical ocular control, resolution of subretinal fluid, stable or improved BCVA, active inflammation), a patient was classified as having met the outcome only if both eyes independently met the corresponding criterion; persistence of subretinal fluid, active inflammation, or BCVA worsening in either eye classified the patient as not having met that outcome.

### Sample size and case ascertainment

Due to the retrospective observational approach, no formal a priori sample size estimate was conducted. All consecutive eligible patients during the research period were included to reduce selection bias and provide a realistic real-world population.

### Inclusion and exclusion criteria

#### Inclusion criteria

Individuals aged 18 years or older, with definite or probable acute ocular VKH disease according to the study-specific two-tier designation, exhibiting active intraocular inflammation (including choroiditis, serous retinal detachment, or panuveitis), who have not received therapy for the present episode, and have a minimum follow-up duration of six months.

#### Exclusion criteria

Advanced cataract; incomplete records (including missing BCVA or OCT data); ocular hypertension; pre-existing glaucoma; prior systemic or periocular corticosteroid therapy for VKH; retinal diseases affecting visual outcomes; use of immunosuppressive or biologic agents at presentation.

### Treatment groups

Treatment assignment was individualized according to institutional practice. Patients presenting predominantly with ocular inflammation and absent or minimal systemic manifestations were preferentially considered for PST/STA therapy, whereas patients with clinically significant neurologic, auditory, or other systemic manifestations, severe bilateral panuveitis, or extensive ocular inflammation were preferentially treated with systemic oral corticosteroids. In patients with contraindications to systemic corticosteroids, such as uncontrolled diabetes mellitus or hypertension, PST/STA was preferred when clinically appropriate. The final treatment decision was made by the treating physician after consideration of disease severity and patient-specific factors.

### Group A: Oral prednisolone

Patients received oral prednisolone 1.0 mg/kg/day (maximum 80 mg/day) for approximately one week, followed by gradual tapering over 3–6 weeks according to the treating physician’s clinical judgment and individual patient response. This abbreviated tapering schedule, rather than the slow taper over a minimum of six months recommended in international VKH literature, was used consistently at our institution throughout the 2021–2025 study period. It reflected a departmental practice pattern of prioritizing rapid initial suppression of inflammation with early transition to close outpatient monitoring, rather than a prolonged systemic corticosteroid course, largely to limit cumulative systemic corticosteroid exposure and its associated metabolic risks in this patient population. It was not a standardized, evidence-based VKH tapering protocol, and this is discussed as an important limitation below. Based on the stated dosing schedule, the estimated cumulative prednisolone exposure was approximately 18–28 mg/kg administered over total treatment duration of 4 to 7 weeks (one week at 1 mg/kg/day followed by a 3- to 6-week linear taper), substantially lower than the cumulative exposure associated with a conventional 6-month taper. Adjunctive topical prednisolone acetate 1% and cyclopentolate 1% were used at the treating physician’s discretion during the acute phase in both groups; the topical regimen and tapering schedule did not differ systematically between treatment groups. Accordingly, the oral prednisolone arm should be considered an abbreviated real-world systemic corticosteroid strategy rather than a conventional prolonged systemic treatment regimen for acute VKH.

### Group B: Posterior sub-Tenon triamcinolone acetonide (PST/STA)

Patients underwent posterior sub-Tenon injection of triamcinolone acetonide (40 mg/1 mL) through the posterior sub-Tenon space. In patients with bilateral disease, bilateral posterior sub-Tenon injections were administered. A repeat injection was performed after 4–6 weeks if persistent ocular inflammation remained. Systemic corticosteroids were reserved for treatment failure. As in Group A, adjunctive topical prednisolone acetate 1% and cyclopentolate 1% were used at the treating physician’s discretion; the topical regimen did not differ systematically between groups.

### Rescue therapy

Defined as initiation of systemic corticosteroids or additional periocular injections due to worsening BCVA by ≥ 2 Snellen lines, persistent or increasing subretinal fluid on OCT, or ongoing inflammation after 2–4 weeks. Rescue events were recorded in the outcome analysis.

### Recurrence definition

Recurrence was defined as the reappearance of clinically active intraocular inflammation (anterior chamber cells/flare or vitreous haze on slit-lamp examination and indirect ophthalmoscopy) and/or new subretinal fluid on OCT, occurring in a patient who had previously achieved and had documented complete inactivity, that is, a patient who had already met the absence-of-inflammation and absence-of-SRF components of the primary outcome definition. Recurrence was confirmed on two consecutive study visits before being recorded as an event. Patients who never achieved complete inactivity during the study period were not classified as having a recurrence, since recurrence by definition requires a prior period of quiescence.

### Data collection and outcome measures

Extracted data included demographics (age, sex), baseline BCVA, OCT findings, treatment modality, follow-up BCVA at 1, 3, and 6 months, recurrence episodes, and adverse events. Spectral-domain OCT (RTVue XR Avanti, Optovue Inc., Fremont, CA, USA) was used at every visit, with a standardized macular volume (“3D Retina”) scan protocol centered on the fovea. Central macular thickness (CMT) was defined as the average retinal thickness within the central 1-mm ETDRS subfield, using the device’s built-in automated retinal-boundary segmentation algorithm; segmentation was reviewed by the examining ophthalmologist at each visit, with manual correction of segmentation errors when present. The degree of intraocular inflammation at baseline was recorded qualitatively in the clinical record but was not captured with a standardized grading instrument and could not be reliably compared between groups; it is therefore not included in Table 1, and this is noted as a limitation.

**Table 1:** Baseline characteristics

Variable	Prednisolone (*n* = 15)	PST/STA (*n* = 15)	*p*-value
Age (years), mean ± SD	36.4 ± 9.1	35.7 ± 8.8	0.82
Male sex, n (%)	9 (60.0%)	8 (53.3%)	1.00
Baseline BCVA (logMAR), mean ± SD	0.78 ± 0.24	0.81 ± 0.27	0.74
Subretinal fluid on baseline OCT, n (%)	15 (100%)	15 (100%)	NA
Optic disc edema, n (%)	10 (66.7%)	11 (73.3%)	1.00
Time from symptom onset to treatment (days), median (IQR)	6 (4–9)	7 (4–10)	0.68

BCVA = best-corrected visual acuity; PST/STA = posterior sub-Tenon triamcinolone acetonide; OCT = optical coherence tomography; IQR = interquartile range; NA = not applicable. Categorical variables were compared using two-sided Fisher’s exact test; continuous variables using the independent t-test or Mann–Whitney U test, as appropriate

#### Primary outcome

Complete clinical and anatomical ocular control at three months, a composite endpoint defined as the simultaneous presence of all three of the following: (1) absence of clinically active intraocular inflammation, defined as absence of anterior chamber cells/flare and vitreous haze on slit-lamp examination and indirect ophthalmoscopy at the study visit, assessed qualitatively by the examining ophthalmologist rather than with a standardized quantitative grading instrument such as the Standardization of Uveitis Nomenclature (SUN) scale; (2) qualitative disappearance of subretinal fluid on OCT, defined as the absence of a visible subretinal fluid pocket at the central fovea and surrounding macula on structural OCT scan; and (3) stable or improved BCVA relative to baseline. Central macular thickness (CMT) was recorded in parallel at each visit as a supportive structural measure of retinal recovery but was not used as a quantitative measure of subretinal fluid volume or height, since CMT reflects both retinal tissue thickness and, when present, the contribution of subretinal fluid, rather than fluid volume alone. Subretinal fluid was not separately quantified by maximum height, area, or volume in this study. OCT-based findings were considered anatomical outcome measures only and were not interpreted as evidence of resolution of choroidal inflammation. Although OCT is not included in the revised international diagnostic criteria for VKH and is not a standardized imaging modality for diagnosis or follow-up, it was used here as an adjunctive, non-invasive tool to monitor anatomical response to treatment over time.

#### Secondary outcomes

BCVA at six months, recurrence rate and time to first recurrence, need for rescue therapy, incidence of steroid-related adverse events, and OCT structural outcomes. Cataract progression was defined as new lens opacities reducing BCVA by ≥ 2 lines; hyperglycemia was defined as fasting blood glucose > 126 mg/dL or new initiation of antidiabetic therapy; hypertension as systolic > 140 mmHg or diastolic > 90 mmHg, or initiation of antihypertensive treatment.

### Statistical analysis

Normality of continuous variables was assessed using the Shapiro–Wilk test. Normally distributed variables were summarized as mean ± standard deviation, and non-normally distributed variables as median with interquartile range (IQR). Categorical variables were expressed as frequencies and percentages. Between-group comparisons were performed using independent t-test or Mann–Whitney U test for continuous variables, and two-sided Fisher’s exact test for categorical variables. Within-group changes in BCVA were assessed with the paired t-test. Recurrence-free survival was estimated using the Kaplan–Meier method and compared with the log-rank test. Analyses were performed using IBM SPSS Statistics (IBM Corp., Armonk, NY, USA), with two-sided *p* < 0.05 considered significant. All categorical comparisons (Tables 1, 2, 4 and 5, and the Results text) were performed using two-sided Fisher’s exact test throughout, given the small cell counts in every comparison. Risk differences with 95% confidence intervals (Newcombe hybrid score method) are additionally reported for the primary outcome and key secondary outcomes, because with 15 patients per group and no prespecified equivalence or non-inferiority design, a non-significant p-value cannot be interpreted as evidence that the two treatment strategies are comparable.

**Table 2:** Primary outcome at 3 months

Outcome	Prednisolone (*n* = 15)	PST/STA (*n* = 15)	*p*-value
Complete clinical and anatomical ocular control, n (%)	12 (80.0%)	11 (73.3%)	1.00
Resolution of subretinal fluid on OCT, n (%)	13 (86.7%)	12 (80.0%)	1.00
Stable or improved BCVA, n (%)	14 (93.3%)	13 (86.7%)	1.00
Persistent active inflammation, n (%)	3 (20.0%)	4 (26.7%)	1.00
Required rescue therapy by 3 months, n (%)	3 (20.0%)	4 (26.7%)	1.00

All p-values calculated using two-sided Fisher’s exact test. Risk differences (prednisolone minus PST/STA) with 95% confidence intervals: complete clinical and anatomical ocular control, 6.7% points (95% CI − 23.0 to 35.1); resolution of subretinal fluid, 6.7% points (95% CI − 21.1 to 33.6); stable or improved BCVA, 6.7% points (95% CI − 18.4 to 31.8); persistent active inflammation, − 6.7% points (95% CI − 35.1 to 23.0); required rescue therapy, − 6.7% points (95% CI − 35.1 to 23.0). Given the small sample size and the absence of a prespecified equivalence or non-inferiority design, non-significant p-values and the wide confidence intervals shown here should not be interpreted as evidence that the two treatments are comparable

**Table 3:** Visual acuity (BCVA) over time

Time point	Prednisolone (*n* = 15) Mean ± SD	PST/STA (*n* = 15) Mean ± SD	*p*-value
Baseline	0.78 ± 0.24	0.81 ± 0.27	0.74
3 months	0.42 ± 0.18	0.45 ± 0.21	0.67
6 months	0.31 ± 0.15	0.34 ± 0.19	0.68

BCVA = best-corrected visual acuity (logMAR). Comparisons by independent t-test. Six-month values include seven patients (23.3%; 3 prednisolone, 4 PST/STA) who received rescue therapy; these results reflect combined initial and rescue treatment and should not be attributed exclusively to the initially selected treatment

**Table 4:** OCT anatomical outcomes

Parameter	Prednisolone (*n* = 15)	PST/STA (*n* = 15)	*p*-value
Baseline CMT (µm), mean ± SD	512 ± 78	518 ± 82	0.78
3 months CMT (µm), mean ± SD	321 ± 45	335 ± 49	0.41
6 months CMT (µm), mean ± SD	298 ± 35	305 ± 38	0.53
Complete subretinal fluid resolution at 6 months, n (%)	14 (93.3%)	13 (86.7%)	1.00

CMT = central macular thickness; OCT = optical coherence tomography. Subretinal fluid resolution was assessed qualitatively. Continuous variables compared by independent t-test; categorical variables by two-sided Fisher’s exact test. Six-month values include seven patients (23.3%; 3 prednisolone, 4 PST/STA) who received rescue therapy; these results reflect combined initial and rescue treatment and should not be attributed exclusively to the initially selected treatment

**Table 5:** Adverse events

Adverse Event	Prednisolone (*n* = 15)	PST/STA (*n* = 15)	*p*-value
Elevated IOP (> 21 mmHg)	2 (13.3%)	3 (20.0%)	1.00
Cataract progression	1 (6.7%)	1 (6.7%)	1.00
Hyperglycemia requiring adjustment	3 (20.0%)	0 (0%)	0.224
Hypertension	2 (13.3%)	0 (0%)	0.483
Local injection site discomfort	0 (0%)	2 (13.3%)	0.483

IOP = intraocular pressure. Hyperglycemia = fasting glucose > 126 mg/dL or initiation of antidiabetic therapy. Hypertension = systolic > 140 mmHg or diastolic > 90 mmHg, or initiation of antihypertensive therapy. All p-values calculated using two-sided Fisher’s exact test. Risk differences (prednisolone minus PST/STA) with 95% CI: elevated IOP, − 6.7% points (95% CI − 33.6 to 21.1); hyperglycemia, 20.0% points (95% CI − 4.2 to 45.2); hypertension, 13.3% points (95% CI − 9.2 to 37.9); local injection-site discomfort, − 13.3% points (95% CI − 37.9 to 9.2)

## Results

### Study population

**Fig. 1:**
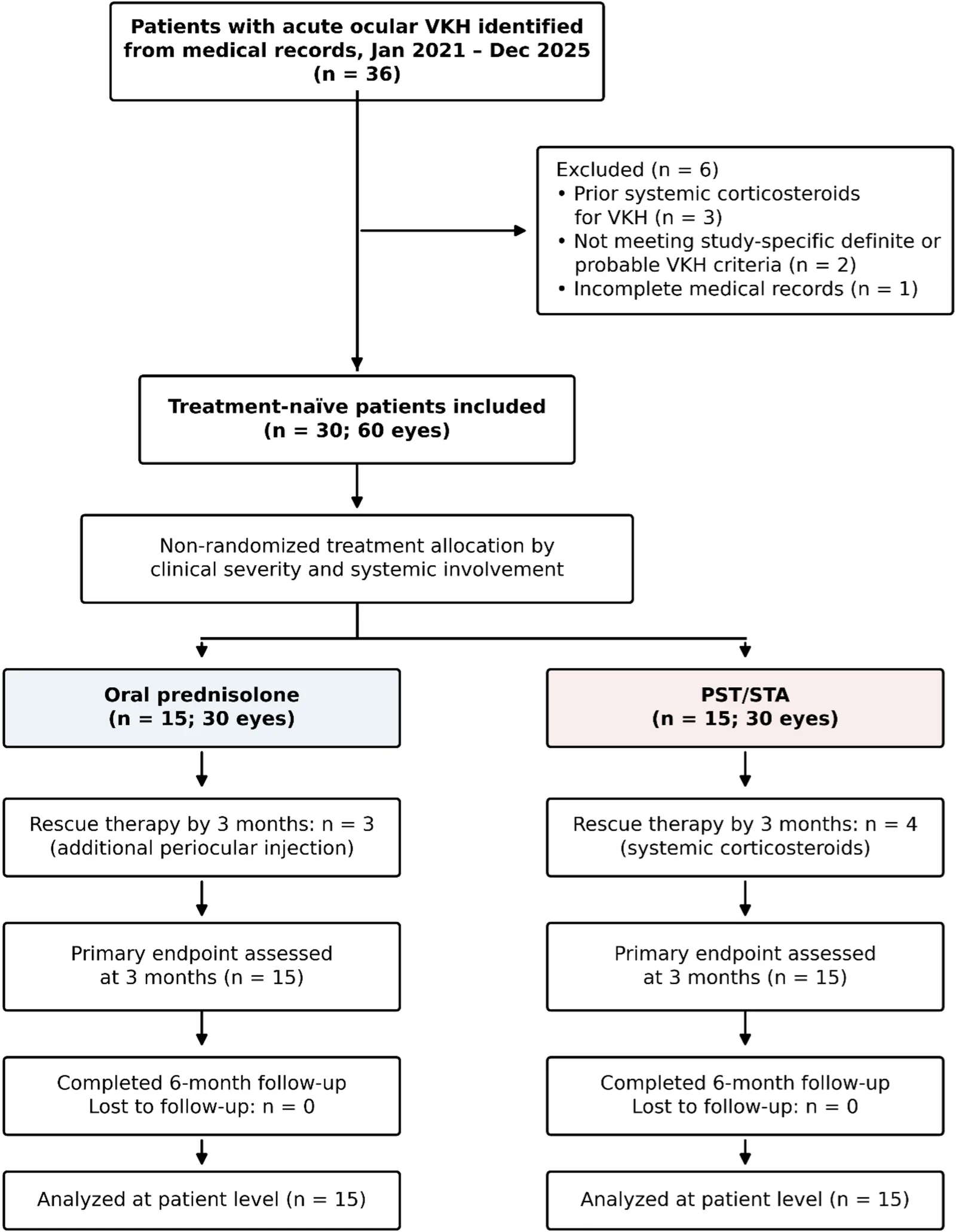
Participant Flow Diagram of Study Selection and Treatment Allocation. Legend: Flowchart illustrating the identification, exclusion, non-randomized treatment allocation and follow-up of patients with acute ocular Vogt–Koyanagi–Harada (VKH) disease. Of 36 patients identified, 6 were excluded: three for prior systemic corticosteroid treatment for VKH, two for not meeting criteria for the study-specific definite or probable VKH designation, and one for incomplete medical records. A total of 30 treatment-naïve patients (60 eyes) were included and allocated to oral prednisolone (*n* = 15; 30 eyes) or posterior sub-Tenon triamcinolone acetonide (PST/STA; *n* = 15; 30 eyes) according to clinical severity and systemic involvement. Rescue therapy was required by 3 months in 3 and 4 patients, respectively, and these patients were classified as not achieving the primary outcome. All 30 patients were assessed for the primary endpoint at 3 months and completed 6 months of follow-up. n = number of patients

During the study period, 36 patients with acute ocular VKH disease were identified. Six patients (16.7%) were excluded: three due to prior systemic corticosteroid treatment, two for not meeting criteria for the study-specific definite or probable VKH designation, and one due to incomplete medical records, leaving 30 patients eligible for analysis (Fig. 1, participant flow diagram). A formal comparison of baseline characteristics between the six excluded and 30 included patients was not performed, as sufficiently complete and standardized baseline data were not available for all excluded patients to permit a reliable comparison. Because complete-case inclusion required a minimum of six months of documented follow-up, patients with better clinic adherence or more favorable early clinical courses may be over-represented in the analyzed cohort, and this is noted as a limitation. Of these, 15 patients received oral prednisolone (Group A) and 15 received PST/STA (Group B). All patients had bilateral involvement, resulting in 60 eyes; analyses were performed at the patient level to avoid statistical dependence between eyes. Patients were classified using the study-specific two-tier designation (definite or probable VKH) derived from the 2001 Revised Diagnostic Criteria, as described in the Methods. Median follow-up was 6.4 months (IQR 6.0–7.1) across the analyzed cohort, with all patients having a minimum of six months of documented follow-up per the inclusion criteria.

### Baseline demographics and clinical characteristics

Baseline demographic and measured ocular characteristics that were assessed showed no significant differences between the two groups (Table 1), including age, sex distribution, baseline best-corrected visual acuity (BCVA), and OCT parameters. However, because treatment assignment was guided by disease severity and systemic involvement rather than randomized, similarity in these specific variables does not establish that the two cohorts had comparable overall disease severity at baseline.

### Primary outcome: clinical and anatomical ocular control at 3 months

Complete clinical and anatomical ocular control at three months (defined as absence of clinically active intraocular inflammation, resolution of subretinal fluid on OCT, and stable or improved BCVA, each required bilaterally) was achieved in 12 of 15 patients (80.0%) in the oral prednisolone group and 11 of 15 patients (73.3%) in the PST/STA group, with no significant difference (*p* = 1.00, Table 2). This primary endpoint was assessed at the 3-month study visit; the three prednisolone-treated and four PST/STA-treated patients who met criteria for rescue therapy by that visit were classified as not having achieved complete clinical and anatomical ocular control, consistent with a treatment-strategy analysis in which rescue is treated as an initial-treatment failure. Accordingly, in Table 2 the number of patients requiring rescue therapy by 3 months equals the number not achieving complete clinical and anatomical ocular control at 3 months in each group.

Resolution of subretinal fluid on OCT, defined as bilateral resolution, occurred in 86.7% and 80.0% of patients, respectively, with no statistically significant between-group difference (*p* = 1.00). Although VKH is primarily a choroidal inflammatory disease, resolution of subretinal fluid on OCT was used as an objective and reproducible marker of retinal anatomical recovery and was not considered definitive evidence of complete resolution of choroidal inflammation. Its use is acknowledged as a study limitation, as OCT is not included in standard VKH diagnostic criteria or validated for follow-up.

### Visual outcomes (BCVA)

**Fig. 2:**
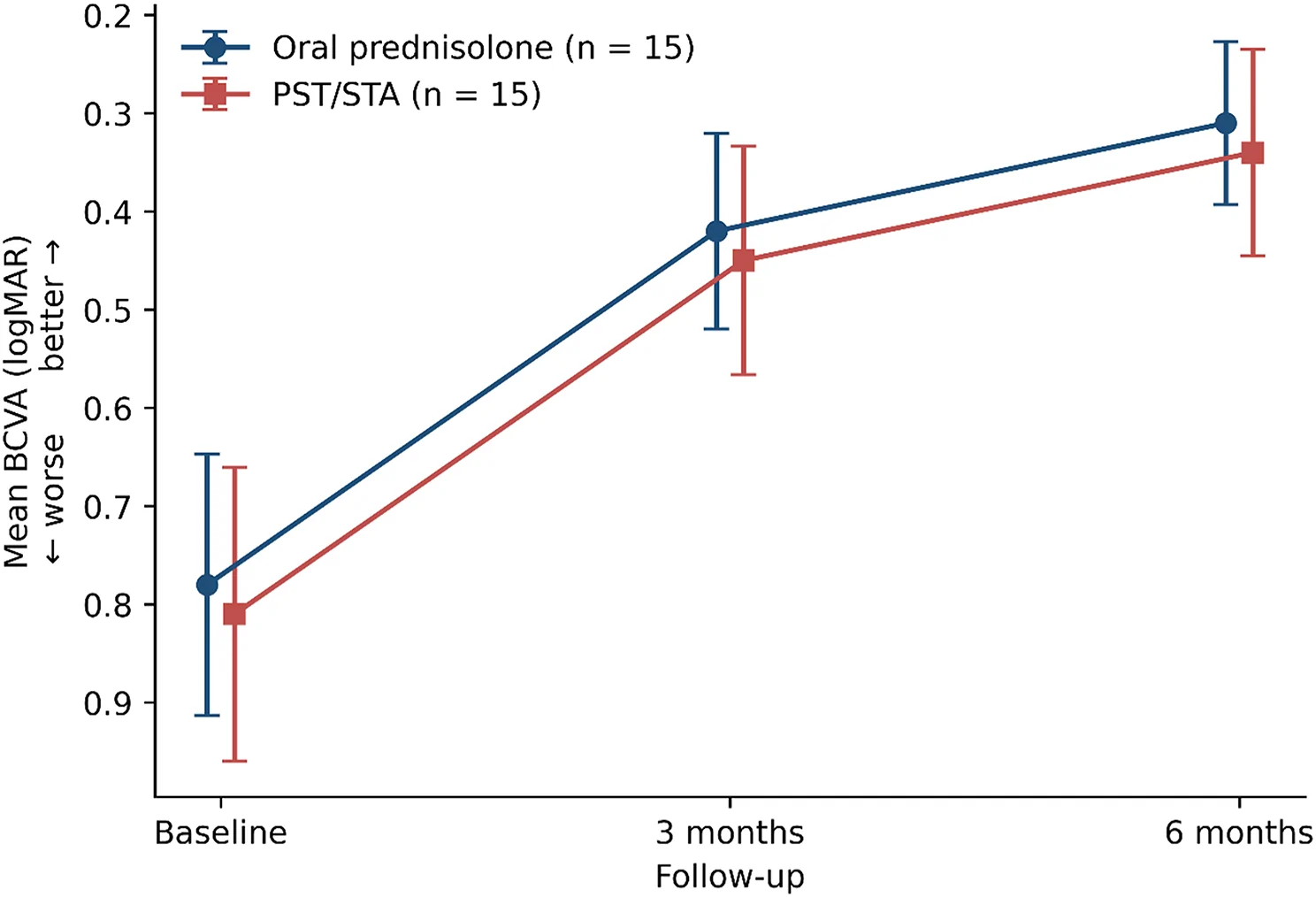
Mean best-corrected visual acuity (BCVA, logMAR) over six months by treatment group. Legend: Lower logMAR values indicate better visual acuity; the y-axis is inverted accordingly. Data points represent group means at baseline, 3 months and 6 months, offset slightly on the x-axis for legibility. Error bars represent 95% confidence intervals calculated from the mean ± SD reported in Table 3 (*n* = 15 per group). Six-month values include seven patients (3 prednisolone, 4 PST/STA) who received rescue therapy

BCVA improved significantly in both groups over six months (*p* < 0.01 for within-group changes, paired t-test). Mean logMAR BCVA at baseline, 3 months, and 6 months are shown in Table 3; Fig. 2. No significant differences were observed between prednisolone and PST/STA groups at any time point.

### OCT outcomes

**Fig. 3:**
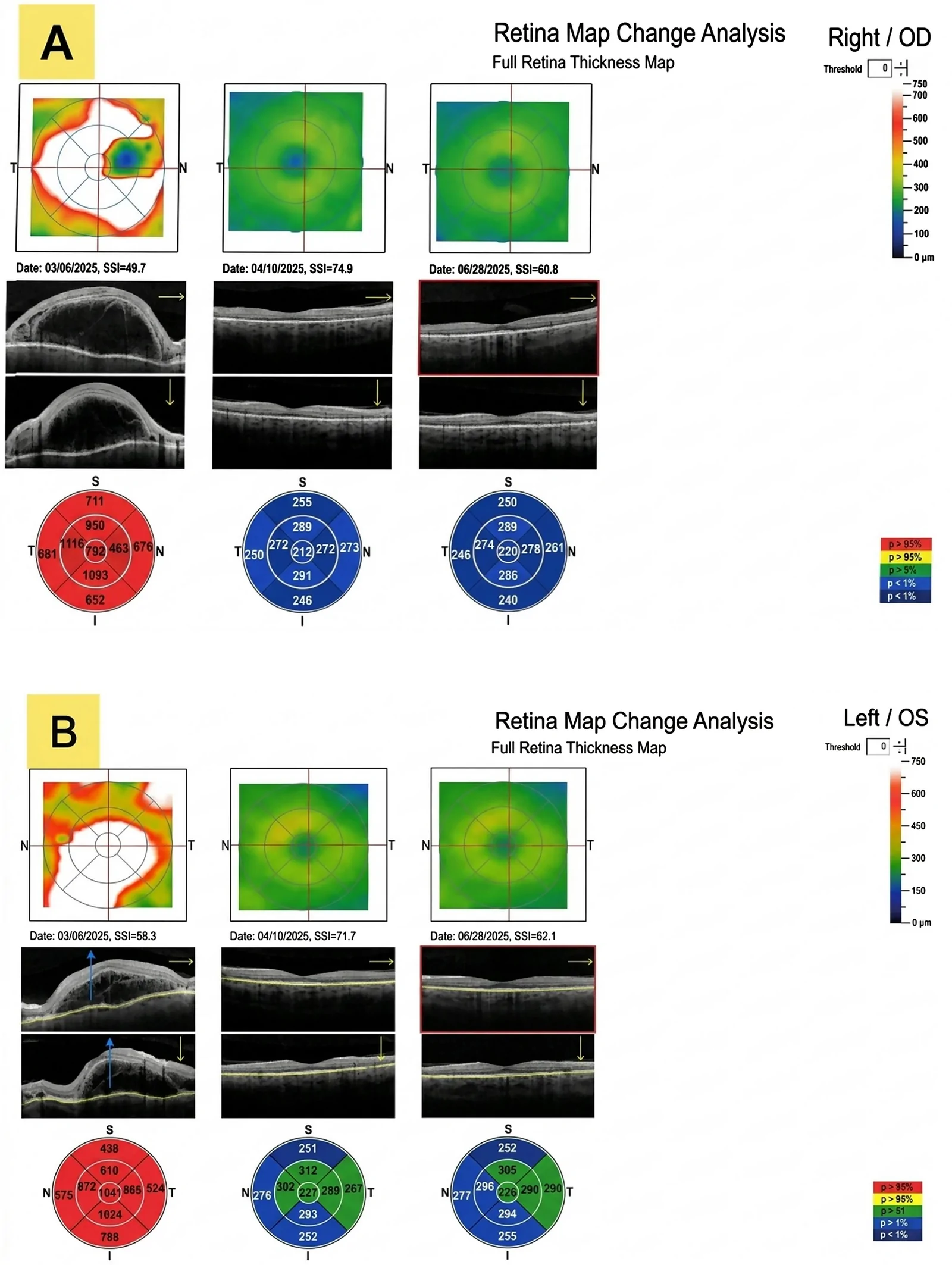
Sequential OCTs in Oral Prednisolone Group (Group A). Legend: Representative spectral-domain optical coherence tomography (OCT) images from a patient with acute Vogt–Koyanagi–Harada (VKH) disease treated with oral prednisolone. **A1**: Right eye before treatment. **A2**: Left eye before treatment, showing serous retinal detachment. **B1**: Right eye after 3 weeks of treatment. **B2**: Left eye after 3 weeks of treatment, showing near-complete resolution of subretinal fluid and restoration of the foveal contour. These images illustrate anatomical recovery in this patient

**Fig. 4:**
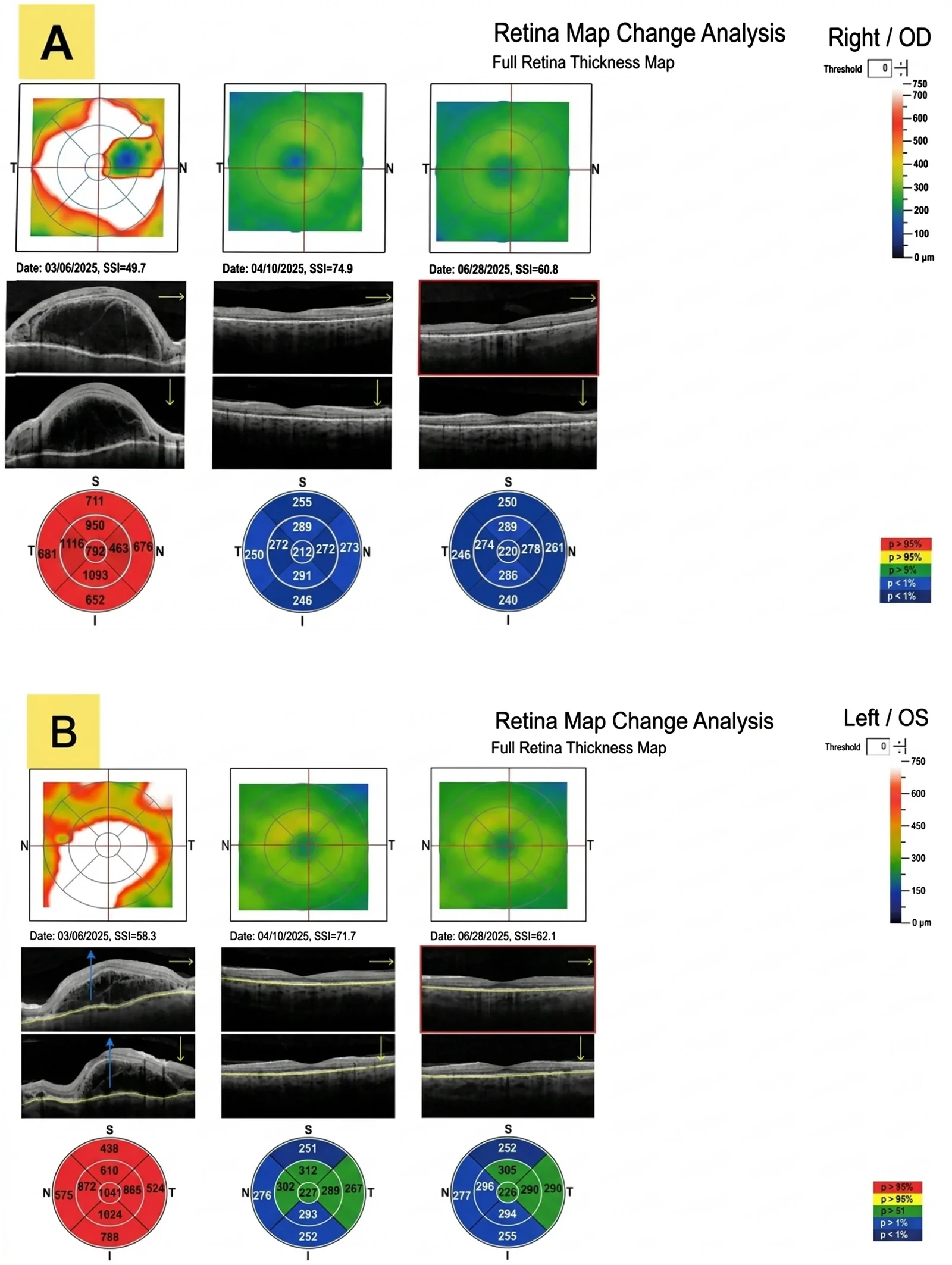
Sequential OCTs in Posterior Sub-Tenon Triamcinolone Acetonide Group (Group B). Legend: Representative spectral-domain optical coherence tomography (OCT) retinal thickness map change analyses in a patient with acute Vogt–Koyanagi–Harada (VKH) disease treated with posterior sub-Tenon triamcinolone acetonide (PST/STA). **A**: Right-eye retinal map change analysis before and after treatment, demonstrating resolution of subretinal fluid and improvement in retinal architecture. **B**: Left-eye retinal map change analysis before and after treatment, similarly demonstrating resolution of subretinal fluid and improvement in the retinal architecture. These sequential retinal map analyses illustrate anatomical recovery in this patient following PST/STA therapy, particularly the resolution of subretinal fluid and restoration of the foveal contour

Both groups demonstrated retinal anatomical improvement, including reduction or resolution of subretinal fluid and improvement in CMT. These findings represent structural retinal recovery and should not be interpreted as definitive evidence of resolution of choroidal inflammation. (Figures 3 and 4; Table 4). Resolution of subretinal fluid was assessed qualitatively on OCT as complete disappearance of the subretinal fluid pocket at the central fovea and surrounding macula; central macular thickness (CMT) was recorded in parallel as a supportive structural measure of retinal recovery rather than as a direct quantitative measure of fluid volume. Because three prednisolone-treated and four PST/STA-treated patients (23% of the cohort) received rescue therapy outside their originally assigned strategy, their 6-month BCVA and OCT outcomes reported in Tables 3 and 4 reflect a mix of the original and rescue treatment exposure rather than the effect of the initial therapy alone. A sensitivity analysis presenting 6-month outcomes separately for rescued and non-rescued patients within each group was not performed, as the group-level summary statistics retained for this retrospective cohort do not permit re-stratification by rescue status; this is stated as a limitation below.

### Recurrence and rescue therapy

**Fig. 5:**
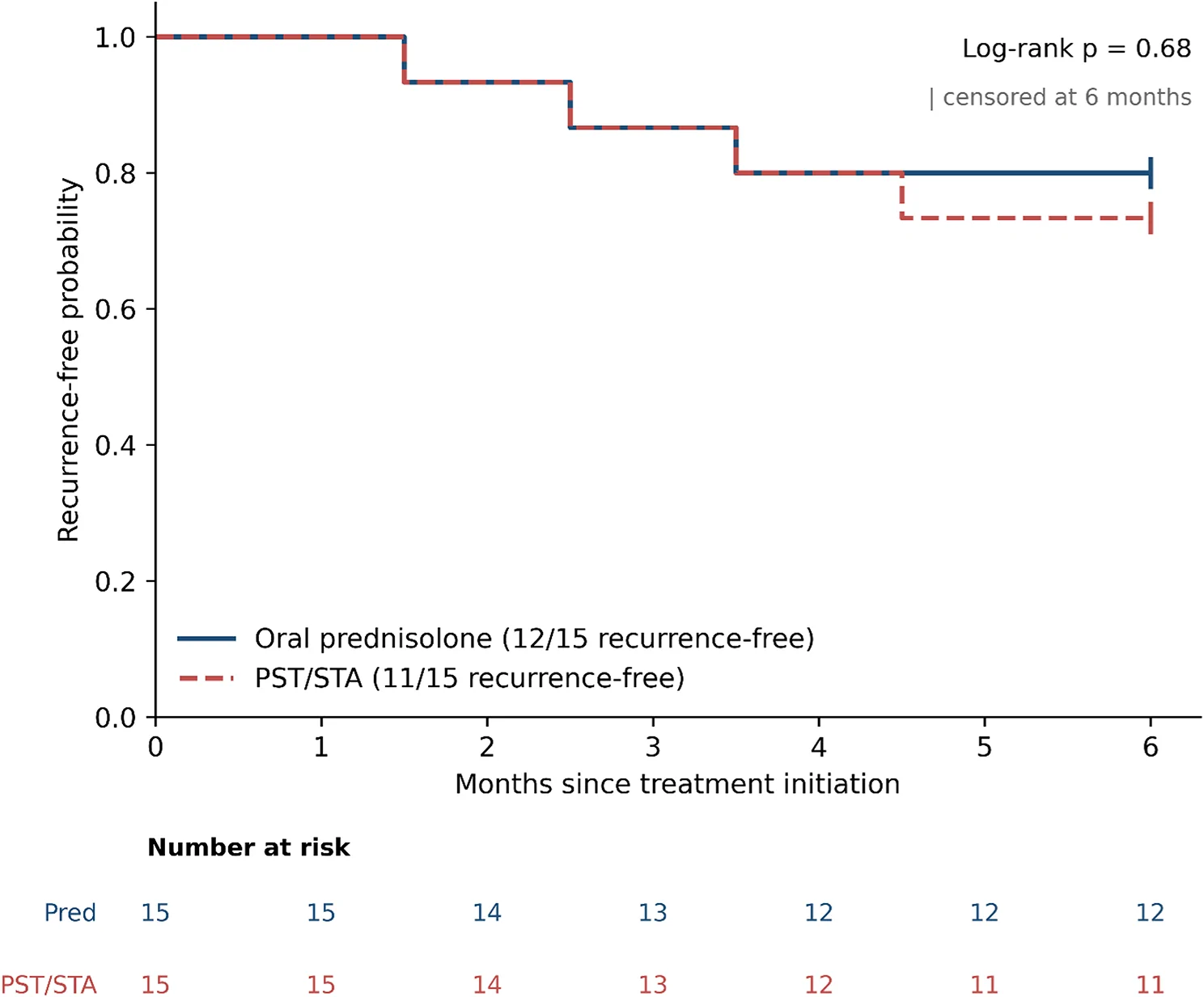
Kaplan–Meier curves for recurrence-free survival during the 6-month follow-up period in the oral prednisolone and posterior sub-Tenon triamcinolone acetonide (PST/STA) groups. Legend: Three of 15 patients (20.0%) in the oral prednisolone group and four of 15 patients (26.7%) in the PST/STA group experienced recurrence during follow-up. There was no statistically significant difference in recurrence-free survival between the groups (log-rank *p* = 0.68), and median recurrence-free survival was not reached in either group. No patient was lost to follow-up; tick marks at 6 months indicate patients censored at the end of the observation period without recurrence. The numbers of patients at risk at each month are shown below the plot

Recurrence occurred in three of 15 patients (20.0%) in the oral prednisolone group and four of 15 patients (26.7%) in the PST/STA group, with no statistically significant difference in recurrence-free survival (log-rank *p* = 0.68; Fig. 5). The numbers of patients at risk at 0, 1, 2, 3, 4, 5, and 6 months were 15, 15, 14, 13, 12, 12, and 12 in the oral prednisolone group and 15, 15, 14, 13, 12, 11, and 11 in the PST/STA group.

Rescue therapy was required in 3 patients (20%) in the prednisolone group (additional periocular injections) and 4 patients (26.7%) in the PST/STA group (systemic steroids), with no statistically significant between-group difference (two-sided Fisher’s exact test, *p* = 1.00). Because rescue therapy was itself an important treatment-strategy endpoint, patients who required rescue are reported in Table 2 as not having achieved the primary 3-month outcome, rather than being excluded from the denominator or counted as successes of their originally assigned treatment. No patient was lost to follow-up before six months; patients who remained recurrence-free were censored at the end of the six-month observation period. The Kaplan–Meier estimates are therefore concordant with the simple six-month recurrence proportions (3/15 vs. 4/15, Fisher’s exact *p* = 1.00). Because fewer than 50% of patients in either group experienced recurrence, median recurrence-free survival was not reached in either group. The six-month recurrence-free proportions were 80.0% in the prednisolone group and 73.3% in the PST/STA group.

### Safety and adverse events

Both treatment modalities were associated with a low frequency of the adverse events assessed during the six-month follow-up period in this small cohort (Table 5); these findings describe events observed during this period only and should not be interpreted as establishing the overall safety of either strategy. Systemic corticosteroid-related adverse events, including hyperglycemia and hypertension, occurred more frequently in the prednisolone group. Baseline diabetes mellitus status, baseline glucose control, baseline hypertension status, and concurrent systemic medications prior to treatment were not systematically extracted for each patient in this retrospective chart review and cannot be reported by group. Because contraindications to systemic corticosteroids, including uncontrolled diabetes or hypertension, influenced treatment allocation, baseline metabolic risk may have differed between groups; the hyperglycemia and hypertension events reported in Table 5 (new-onset or requiring treatment initiation/adjustment during follow-up) therefore cannot be fully distinguished from progression of pre-existing, unreported metabolic disease, and the between-group comparison of these events should be interpreted with this in mind. The number of posterior sub-Tenon triamcinolone acetonide injections received by each individual patient in the PST/STA group was not retained at the per-patient level in this retrospective review and cannot be reported patient-by-patient; per the treatment protocol described in the Methods, a repeat injection was administered at 4–6 weeks only in patients with persistent ocular inflammation, so patients in this group received either one or two injections during the study period. Local injection-related discomfort was observed only in the PST/STA group. No serious ocular complications, such as endophthalmitis or retinal detachment, were reported. Cumulative systemic corticosteroid exposure after rescue therapy was not calculated separately for the patients who received rescue treatment (three prednisolone-treated patients who received additional periocular injections, and four PST/STA-treated patients who received systemic corticosteroids); this additional exposure is not reflected in the cumulative dose estimate reported in the Methods for the original prednisolone regimen, and its absence is noted as a limitation below.

### Immunomodulatory therapy

None of the included patients received systemic immunomodulatory therapy, either at baseline or during the 6-month follow-up period. Therefore, all observed clinical and anatomical outcomes reflected corticosteroid-based treatment alone, without the potential confounding effect of adjunctive immunomodulatory agents.

## Discussion

In this retrospective, non-randomized cohort study, no statistically significant differences were detected between oral prednisolone and posterior sub-Tenon triamcinolone acetonide (PST/STA) in clinical and anatomical ocular control, visual acuity, OCT outcomes, or recurrence over six months. Treatment assignment was guided by baseline disease severity and systemic involvement rather than randomized, and the two strategies therefore cannot be considered comparable in efficacy; the findings instead describe the real-world experience with each strategy at our center in patients who were selected for that strategy based on their disease characteristics. Because PST/STA provides localized corticosteroid delivery, it should not be regarded as a substitute for systemic therapy in patients with significant extraocular manifestations of VKH. Although the cohort was classified using a study-specific two-tier (definite/probable) designation derived from the 2001 Revised Diagnostic Criteria, contemporary VKH literature increasingly emphasizes disease stage, particularly the distinction between acute initial-onset and chronic recurrent disease, because stage has important therapeutic and prognostic implications. Our retrospective dataset did not use a formal contemporary staging framework at the time of treatment allocation. This limits direct comparison with studies specifically designed around stage-based treatment strategies.

Systemic corticosteroids remain the cornerstone of acute VKH management, aiming to suppress the T cell–mediated inflammation that drives multisystem involvement. Early, high-dose therapy followed by a slow taper over at least six months is recommended to reduce recurrences and chronic complications, including sunset-glow fundus, cataract, and glaucoma. Shorter courses or rapid tapering are associated with higher relapse rates [9]. The 3- to 6-week taper used at our institution throughout the study period departs from this recommendation and reflects a departmental practice pattern that prioritized minimizing cumulative systemic corticosteroid exposure and its metabolic risks over prolonged systemic immunosuppression; its implications are considered with the study limitations below.

OCT-based assessment of subretinal fluid was used as a marker of retinal anatomical recovery; as discussed below, it cannot establish resolution of choroidal inflammation, and these findings should be interpreted as supportive anatomical measures only.

Recurrence occurred in 20.0% of the prednisolone group and 26.7% of the PST/STA group during six months of follow-up; median recurrence-free survival was not reached in either group. These findings are consistent with literature showing that relapses often occur within the first 6–12 months, particularly when systemic corticosteroids are tapered without adjunctive immunomodulatory therapy. The absence of immunomodulatory therapy in this cohort is discussed as a limitation below.

Substantial anatomical improvement was observed in both treatment groups. The rates of complete clinical and anatomical ocular control at three months were 80.0% and 73.3% in the prednisolone and PST/STA groups, respectively. These findings can be considered in the context of the earlier observational study by Hosoda et al., who reported complete resolution without recurrence in 21 of 27 eyes (78%) treated with primary PST/STA in acute VKH. However, differences in study design, patient selection, treatment protocols, and outcome definitions limit direct comparison [4]. The use of PST/STA minimizes systemic corticosteroid exposure, which is clinically relevant given the metabolic and immunologic side effects of high-dose oral therapy.

Systemic corticosteroid-related metabolic events (hyperglycemia and hypertension) were observed only in the prednisolone group, whereas PST/STA was associated with transient local discomfort and mild intraocular pressure elevation. No serious ocular complications occurred. As discussed below, these observations cannot be firmly attributed to treatment toxicity because baseline metabolic status was not available by group.

This study has important limitations that should be considered when interpreting its findings. The retrospective, non-randomized design and relatively small sample size (15 patients per treatment group) limit the strength and generalizability of the comparative conclusions. Treatment allocation was influenced by clinical presentation and physician preference, resulting in potential confounding by indication. PST/STA was preferentially used in patients with predominantly ocular, non-severe disease and absent or minimal systemic manifestations, whereas oral prednisolone was more often selected for patients with clinically significant neurologic, auditory, or integumentary manifestations, severe bilateral panuveitis, extensive ocular inflammation, or contraindications to systemic corticosteroids. Detailed baseline information regarding diagnostic subclassification, systemic disease severity, standardized anterior chamber and vitreous inflammatory grades, extent of serous retinal detachment, optic disc involvement, and baseline metabolic risk factors was not consistently available in the retrospective records. The clinical reasoning underlying individual treatment decisions was also not systematically documented. Consequently, residual and unmeasured confounding could not be adequately controlled, and the treatment groups should not be considered directly comparable in terms of baseline disease severity.

The relatively small sample size also provided limited statistical power, and only seven recurrence events occurred during follow-up, restricting the precision of recurrence-rate estimates and Kaplan–Meier analyses. The statistical analyses were designed to test for differences between groups and were not designed or powered to demonstrate equivalence or non-inferiority; no equivalence or non-inferiority margin was prespecified. BCVA and CMT were compared cross-sectionally at each visit, and no mixed-effects model with a group-by-time interaction was fitted, so differences in the rate of change between groups were not formally tested. Therefore, non-significant p-values should not be interpreted as evidence of comparable efficacy. The wide confidence intervals around the estimated between-group differences, including those for the primary outcome, indicate that clinically meaningful differences in either direction cannot be excluded.

The six-month follow-up period is another important limitation. Although this duration allows assessment of early treatment response and short-term recurrence, it is insufficient to fully capture late recurrences, progression to chronic recurrent disease, or the development of sunset-glow fundus, which may occur over a substantially longer time course. The limited number of recurrence events further restricts the precision with which long-term recurrence risk can be estimated.

The corticosteroid regimen used in the oral prednisolone group was also shorter than contemporary prolonged tapering strategies recommended for initial-onset VKH. Patients received an approximately 18–28 mg/kg cumulative dose over 4–7 weeks, reflecting the departmental practice during the study period and an effort to limit cumulative systemic corticosteroid exposure rather than a standardized evidence-based long-term taper. Because prolonged corticosteroid tapering has been associated with a lower risk of recurrence, the recurrence rate observed in the prednisolone group may differ from that expected with a conventional prolonged taper. Recent studies have also examined different systemic corticosteroid regimens in VKH, further emphasizing that treatment intensity and duration may influence outcomes [10]. Accordingly, the present study compares PST/STA with an abbreviated systemic corticosteroid strategy and should not be interpreted as a comparison with standard contemporary systemic treatment.

Another important limitation is the absence of immunomodulatory therapy (IMT) as part of the initial treatment strategy. During the study period, none of the patients received IMT at presentation, reflecting institutional practice and physician preference rather than a predefined study protocol. Although early and risk-stratified use of IMT is increasingly recommended in selected patients with acute initial-onset VKH to improve long-term disease control and reduce the risk of chronic recurrence [11], our study was designed to compare two corticosteroid-based treatment strategies used in routine clinical practice. Because the present cohort did not receive IMT and used a shorter systemic corticosteroid taper than currently recommended, our findings should be interpreted within this evolving treatment paradigm and should not be considered representative of contemporary best-practice VKH management. In particular, the findings should not be interpreted as supporting corticosteroid monotherapy as the preferred contemporary treatment strategy.

An additional limitation concerns anatomical assessment. Structural OCT was used to evaluate subretinal fluid resolution and central macular thickness; however, OCT primarily reflects retinal structural recovery and does not by itself establish complete resolution of choroidal inflammation. Enhanced-depth imaging or swept-source OCT measurements of subfoveal choroidal thickness were not routinely performed during the study period, and indocyanine green angiography (ICGA) was not systematically performed. Consequently, persistent or subclinical choroidal inflammatory activity could not be assessed longitudinally using dedicated choroidal imaging modalities. In line with recent consensus recommendations emphasizing multimodal imaging in VKH, the absence of quantitative choroidal thickness measurements and ICGA is an important limitation of the anatomical assessment [6]. Resolution of subretinal fluid and normalization of central macular thickness on structural OCT indicate retinal recovery but should not be equated with complete resolution of choroidal inflammation. Recent work has further highlighted the potential value of early structural and choroidal imaging biomarkers in monitoring treatment response in VKH [12].

A related limitation is that ocular inflammatory activity was assessed qualitatively by the examining ophthalmologist rather than using a standardized quantitative grading system such as the SUN criteria. Similarly, subretinal fluid resolution was determined qualitatively as present or absent on OCT rather than by quantitative measurement of fluid height, area, or volume. These limitations may have reduced the precision and reproducibility of outcome assessment.

Because of the retrospective nature of the study, extraocular manifestations were not consistently documented with sufficient detail to reliably classify all patients according to the complete three-tier 2001 Revised Diagnostic Criteria for VKH. We therefore used a two-tier definite/probable classification based on the available documentation. Systemic manifestations were also not systematically graded using a standardized severity scale. This limits the ability to fully characterize baseline systemic disease severity and restricts comparison with studies using the complete three-tier diagnostic classification and standardized systemic severity assessment.

Rescue therapy represents an additional limitation. Three patients in the oral prednisolone group and four patients in the PST/STA group required rescue therapy within three months and were classified as not achieving the primary outcome according to the treatment-strategy analysis. However, the six-month BCVA and OCT outcomes include both rescued and non-rescued patients. Therefore, these longer-term outcomes represent mixed treatment exposure in a substantial minority of patients and cannot be attributed exclusively to the initially assigned treatment strategy. A sensitivity analysis excluding rescued patients was not performed because of the retrospective design and limited sample size. Future prospective studies should therefore incorporate predefined rescue criteria and per-patient outcome tracking according to rescue status.

Interpretation of the safety outcomes is also limited by incomplete baseline information. Contraindications or concerns regarding systemic corticosteroid use, including diabetes and hypertension, influenced treatment selection; however, baseline diabetes, glucose status, hypertension, and concurrent systemic medications were not consistently retained or available by treatment group. Consequently, differences in hyperglycemia or hypertension observed during follow-up may partly reflect differences in baseline metabolic risk rather than treatment-related toxicity. Furthermore, individual PST/STA injection numbers and cumulative systemic corticosteroid exposure following rescue therapy were not consistently available. The reported safety outcomes therefore describe adverse events observed during six months in this specific cohort and should not be interpreted as establishing the comparative safety of the two treatment strategies.

Six of the 36 identified patients were excluded before analysis for the reasons described in the Results section and participant flow diagram (Fig. 1). Because sufficiently complete and standardized baseline data were not available for all excluded patients, a reliable comparison between included and excluded patients was not possible. This introduces a potential source of selection bias that should also be considered when interpreting the results.

Overall, these limitations mean that the findings should be regarded as a descriptive real-world comparison of two corticosteroid-based treatment strategies rather than definitive evidence of comparative efficacy, equivalence, or superiority. Larger prospective, multicenter studies with standardized baseline disease-severity assessment, multimodal imaging including quantitative choroidal measurements and ICGA, longer follow-up, predefined rescue criteria, systematic recording of cumulative corticosteroid exposure, and prespecified criteria for early IMT are needed to determine the optimal treatment strategy and long-term outcomes of localized versus systemic corticosteroid therapy in acute VKH.

## Conclusions

In this retrospective, non-randomized cohort, no statistically significant differences were detected between oral prednisolone and posterior sub-Tenon triamcinolone acetonide in the short-term clinical and anatomical outcomes assessed. These findings should be interpreted cautiously because treatment allocation was influenced by baseline disease severity and systemic involvement, important severity determinants were not captured in a sufficiently standardized manner, the sample size was small, and no prespecified equivalence or non-inferiority analysis was performed. The results therefore represent descriptive observations from two differently selected real-world treatment strategies rather than evidence of therapeutic equivalence or comparable efficacy. These findings suggest that localized corticosteroid therapy may have a role in carefully selected patients with predominantly ocular disease and limited systemic involvement, particularly when minimizing systemic corticosteroid exposure is clinically desirable. However, the study does not establish equivalence with contemporary treatment strategies incorporating prolonged corticosteroid tapering and early immunomodulatory therapy. Longer-term prospective studies using standardized multimodal imaging, disease-stage classification, and predefined immunomodulatory treatment strategies are needed to determine the appropriate role of localized corticosteroid therapy in modern VKH management.

## Data Availability

The datasets used and/or analysed during the current study are available from the corresponding author on reasonable request.

## Abbreviations

BCVA: Best-Corrected Visual Acuity
CMT: Central Macular Thickness
IMT: Immunomodulatory Therapy
IOP: Intraocular Pressure
OCT: Optical Coherence Tomography
PST/STA: Posterior Sub-Tenon Triamcinolone Acetonide
SD: Standard Deviation
IQR: Interquartile Range
logMAR: Logarithm of the Minimum Angle of Resolution
VKH: Vogt–Koyanagi–Harada disease
